# Supplementary material for: Are carbon emissions trading and green financial instruments synergistic? -Comprehensive quantitative research based on content analysis
Source: PLoS One. 2024 Mar 7;19(3):e0298601. doi: 10.1371/journal.pone.0298601 (PMC10919601; doi:10.1371/journal.pone.0298601)
Supplement: S1 Data — (ZIP) [file pone.0298601.s001.zip › Data/地级市Z_F工作报告.zip!/┴╔─■/▒╛╧¬╩╨/└┤╫╘╣1⁄2╓┌║┼í╛┬φ┐╦╩2╛▌═°í┐_2015.docx]

各位代表：

现在，我代表本溪市人民政府向大会报告工作，请予审议，并请市政协委员提出意见和建议。

一、2014年工作回顾

过去的一年，在市委的坚强领导下，全市上下积极应对严峻复杂的经济形势，认真落实市十五届人大二次会议确定的目标任务，全市经济社会实现持续稳定发展，各项主要指标增速保持在合理区间、高于全省平均水平。预计：全市生产总值完成1227亿元，同比增长6%；固定资产投资885.2亿元，增长5%；规模以上工业增加值完成570亿元，增长6%；社会消费品零售总额实现333.6亿元，增长12.2%；城镇常住居民人均可支配收入25690元，增长9%，农村常住居民人均可支配收入11740元，增长10%。

一年来，市政府主要做了以下工作：

（一）突出抓好财税收入和项目建设，稳增长取得成效

以财税收入为重点，保持财政收支平稳运行。落实倒逼机制和征收目标责任制，强化收入征管和非税收入监管，促进了财政收入持续稳定增长。全年公共财政预算收入129.8亿元，增长0.1%，是全省6个实现正增长城市之一。通过加强财政预算安排和执行管理、严格实行零基预算、全面公开预决算、健全和完善政府债务偿还机制、加大政府采购和投资审核力度等措施，实现了保工资、保运转，保障了刚性支出和重大项目、民生工程的资金需求。

以项目建设为重点，深入开展项目年活动。采取媒体公示、按月调度、定期考评等方式，落实“点对点”办公、“县区服务日”等推进机制，突出问题导向，推进了全市436个重点项目。丹阜高速石桥子互通立交改造、丹霍线千金岭隧道及引线、新建本溪火车站等一批项目竣工；沈丹客专、田桓铁路、观音阁输水工程等在建项目进展顺利；本桓高速公路等重大项目前期工作取得实质性进展。

以贯彻国务院28号文件精神为统领，抢抓新一轮东北振兴机遇取得进展。在全力以赴跑国家部委、全面覆盖争取省本级政策、全员上阵承担争政策职责上下功夫，积极向政策、向市场、向环境要机遇。溪湖区老工业区搬迁改造、南芬区独立工矿区改造搬迁纳入首批国家政策支持范围；观音阁输水工程纳入国务院支持的东北地区139个在建重大项目名录；市城投三期债、本溪热电联产项目分别获得国家发改委批准、核准；争取省政府出台了支持本溪生物医药产业可持续发展的19条政策措施。抓住“央企辽宁行、知名民企辽宁行、沪企辽宁行”和“著名药企药都行”等活动契机，主动对接35家央企、14家知名民企、27家沪企和30余家国内医药百强企业，推动并对接项目115个，中国供销集团、上海医药集团等企业在溪投资项目正式签约。同时，启动了“十三五”规划编制工作。

（二）打响药都建设总攻战，“四个规模”取得阶段性成果

一是企业规模不断扩大。东北制药辽宁生物、成大动物药业等22户企业实现新投产，累计投产药业企业达到75户；韩国大熊药业等14户企业新开工建设；引进上海绿谷等区域总部及销售类项目5个、产业类项目29个。发挥科技支撑和科技金融作用，药都创新园新引进10个科研团队，推进了50个创新品种的研发。药都创业园建立城市运行管理中心，与辽宁科技学院、神州数码集团共建大学生创业平台。药都已注册6家基金管理公司，总规模7亿元的2支基金进入实质操作阶段，科技融资担保公司开始运营。二是经济规模持续增长。预计高新区完成地区生产总值109亿元，增长9%；公共财政预算收入19.1亿元，增长2.3%；固定资产投资208亿元，增长5%；药业销售收入实现150亿元。三是城市规模快速提升。沈阳药科大学二期工程完成40%；中国医科大学本溪校区二期工程主体封顶；辽宁中医药大学本溪校区和辽宁医药职业学院开工建设；省实验学校本溪分校一期投入使用。推进实施了路网、广场等33项基础设施项目，沈本新城高铁站、客运枢纽站、公安业务技术用房等项目全面开工，完成丹阜高速桃仙机场至石桥子段33.8公里亮化工程。实施了城市空间优化等“六大行动”。教育、健康养生、温泉等特色地产在建项目22个，建筑面积155万平方米。四是人口规模显著增加。目前药都已集聚人口11万人，其中药企从业人员1.5万人，高校入驻师生2.1万人。站在“四个规模”的新起点上，药都建设得到了省委、省政府的高度关注。正如李希省长来本溪调研时所指出，药都走出了很有特点的产城融合建设新城道路，在全省也是一个很好的示范。

（三）加快建设绿色钢都，工业经济发展再上新台阶

按照“1+9+1”的思路抓好工业经济运行。进一步实施“163”绿色钢都战略。全力服务并解决了18个制约本钢发展的问题。本钢集团调整品种结构，内部降耗挖潜，实现销售收入、利润、出口正增长，对全市经济平稳运行起到积极的支撑作用。钢材深加工产业项目进展较快，总投资120亿元的思山岭铁矿开工和新建6条竖井；总投资240亿元的大台沟铁矿启动建设1号坑探矿井；总投资76亿元的本钢冷轧高强钢项目进入调试阶段；由世界焊材产销量第一的天津金桥焊材集团投资11亿元建设的本溪金桥项目实现当年投产。落实工业“五项工程”。新增销售收入10亿元以上企业3户，累计达到16户；工业固定资产投资410亿元，405个工业项目开工建设；全市工业产业集群实现销售收入1050亿元，增长25%；淘汰6座高炉、2座焦炉的落后产能。智慧城市建设全面启动。本溪成为东北地区首个集信息惠民、宽带中国、信息消费三个国家试点（示范）城市于一身的地级市；以市民网和市民卡为载体的市民融合服务平台正式上线发布；云计算数据中心等项目开工建设，为智慧城市建设奠定了坚实基础。

（四）积极建设枫叶之都，旅游产业取得新进展

一是推进项目建设。深入实施温泉旅游“512”战略，温泉寺天著温泉小镇完成综合楼和商务酒店主体施工，汤沟国际温泉旅游度假区花溪沐温泉会馆一期投入运营。我市被国土资源部正式授牌为“中国温泉之城”。铁刹山整体开发、枫林谷二期、关东旅游民俗文化村等项目完工并对外运营。本溪县、桓仁县分别新建、续建20个特色旅游山庄和22个冰酒酒庄。二是加大旅游招商力度。总投资16亿元的北京万景集团铁刹山养老养生项目签约落地，中国国旅、中文时代、万呈集团等旅游项目正在积极洽谈；包装推介30个沟域旅游项目，9个项目实现签约。三是加强宣传营销。整合宣传资源，锁定目标市场，借助央视媒体、智慧旅游等多种形式进行精准营销，有效提升了本溪旅游知名度和影响力。探索设立旅游景区“枫红指数”，促进了赏枫旅游向标准化发展。全年旅游总收入增长16.4%。

（五）认真落实服务业发展《四年行动计划》，重点产业得到发展

房地产业从规范市场入手，引导开发需求导向型的特色地产。全年房地产投资112.6亿元，新开工444.5万平方米，竣工420.8万平方米；商品房销售额130亿元、销售面积329.3万平方米。胜利路三角地开发确定了实施方案，平山甲楼改造启动了征迁工作。商贸物流业编制完成了产业总体发展战略规划；本溪钢都商贸物流产业园区、本溪桥北物流园区被命名为省级物流示范园区；正远物流大厦入驻商贸企业70户；本溪市交通物流中心、桓仁县东北参茸城等项目进展顺利。金融业实现“三个一”的历史性突破，全市银行新增贷款突破100亿元；城商行贷款余额突破100亿元；金融业实现税收突破10亿元，增长72.4%。引进和设立金融机构6家。首家股份制农村商业银行在桓仁正式营业。本钢集团投资10亿元成立本钢财务公司，投资1亿美元成立全省首家外资融资租赁公司。辽宁实华集团在香港主板上市。成功举办了首届金融文化节。电子商务业开始起步，与京东商城、神州数码集团建设了“活力本溪”电子商务平台等项目，推动了一批电商网站上线运营。商贸流通业继续开展各类主题促销活动，保障了商品市场稳定供应。组织开展美食节、啤酒节等“夜经济”主题活动，丰富了市民群众的夜间生活。城区经济突出抓好服务业集聚区建设，平山路地下时尚广场、万鑫国际酒店等项目主体竣工，辽砚文化旅游集聚区成为我市第4个省级现代服务业集聚区。全市服务业集聚区实现主营业务收入312亿元，增长10%。四个城区平均生产总值增长8.4%。

（六）注重抓好县域经济，农业农村工作稳步发展

“一县一业”继续提质增效，本溪县刺五加道地中药材种植面积达到2万亩；桓仁县以冰葡萄为主的酿酒原料基地达到7万亩，成为“国家创建知名品牌（冰酒）示范区”。两县工业产业集群加速发展，本溪县人参铁加工和玻璃制品产业集群实现产值125亿元，桓仁县葡萄酒和包装印刷产业集群累计投产项目达到109个。两县县均生产总值增长8.3%。同时，克服夏旱影响，全年粮食总产量24.4万吨，蔬菜产量21.6万吨，山野菜种植面积达到6.7万亩。全面启动了农村土地确权登记试点。全市新开发林下经济18.8万亩，建设“千万亩经济林”工程4.2万亩。积极开展农田水利建设，新建小型农田水利工程75项，新修、加固河道堤防120公里。完成农村饮水工程41处，解决了2万人饮水安全问题。

（七）深入推进改革开放，发展活力有效增强

加快行政审批制度改革，首批取消和下放行政职权248项，减少行政审批事项59项；通过整合流程、一门受理、并联审批、限时办结，将建设工程项目审批时限由230余天压缩为39天。推进公共行政“一站式”审批服务和“四零”服务工作法并取得较好效果。深化政府机构改革，完成工商、质监行政体制调整，整合加强了交通运输、食品药品监管等领域和部门的职能机构。事业单位分类改革有序推动。工商登记制度改革全面落实，全年新注册登记各类市场主体9906户，增长13.8%，其中公司企业类1540户，增长64.5%。华润医药集团成功重组市医药总公司。大力推进对外开放。继续坚持扁平化、矩阵式投资促进推进体制，市政府班子成员和各县区、各部门分层推动、共同落实对外开放目标。全年围绕生物医药、工业产业集群、服务业集聚区等专题，先后赴京津冀、长三角、珠三角等地以及澳大利亚、韩国、香港、台湾等国家和地区开展“走出去”活动26次，点对点洽谈推进项目102个。各县区自主开展投资促进活动150次。本溪县上海州绿商贸城、明山区同元集团辽东古镇等43个项目签约或开工建设。全年实际利用外资6亿美元，增长16.6%；出口创汇31亿美元，增长15%。引进省外投资到位资金321亿元。辽宁出入境检验检疫局本溪办事处挂牌成立，对外开放服务功能进一步提升。

（八）大力建设生态宜居城市，城乡环境持续改善

城市规划、建设和管理水平继续提升。以“创卫”为抓手，开展市容环境综合整治。重点整治了城市主干路两侧“五乱”以及占道经营、车辆带泥上路等违章行为，依法拆除50个小区和59条街巷路两侧3.28万平方米的违法建筑。溪湖区发挥示范作用，多措并举拆除小区内各类违建1000余处、万余平方米。实施了园林绿化提升工程，在主城区新建11处微广场，清理望溪公园、儿童乐园内违章建筑1.2万平方米。完成“省模”复核验收，正式成为省级环保模范城。新增国家级生态乡镇4个、省级生态乡镇和生态村24个。以农村环境治理为主线建设宜居乡村。整合资金3.45亿元，实施村屯道路硬化、绿化、亮化、垃圾和污水处理等工程，完成11个乡镇、102个行政村的治理任务。

“三大工程”建设取得新成效。青山工程完成超坡地还林2.2万亩，围栏封育8.9万亩，植树造林4万亩。碧水工程完成市污水处理厂提标改造扩容工程，市区生活污水处理能力提高到90%以上，桓仁水库纳入省级水源地保护区综合治理范围。蓝天工程实施了56个重点空气污染治理项目，完成污染物排放总量控制指标任务，全年空气环境质量二级以上良好天数按省政府考核标准达到321天。

（九）顺利完成十大类民生工程，各项社会事业取得进步

一是全年城镇实名制就业6.98万人，城镇登记失业率控制在3.94%以内。二是完成了省下达的社会保险扩面指标。企业退休人员月人均增加养老金186.6元；争取省以上专项资金15.6亿元，确保了企业离退休人员养老金按时足额发放。城乡低保和农村五保供养标准平均提高10%，做到了应保尽保。三是棚户区改造新建15053套安置房。四是新建、改造、续建城市道路12条；北地跨线桥跨河段建成通车；完成110条街巷路改造；更新改造20万平方米人行道；建设村内道路252公里。开通沈本新城榆林华苑至歪头山等8条公交线路。五是在全市推行物业管理权力下放，明山区加强物业管理试点取得成效。县区共投资4600万元整治老旧住宅区、消除34个弃管项目，65个小区实施了自助式物业管理。完成“暖房子”工程243万平方米，同步实施了62个旧住宅区环境配套整治工程。实施主城区农贸市场改造升级11家。六是完成3万户未验收房屋确权工作，平山区在推进确权工作中发挥了示范作用。建设停车场31个，提供停车泊位1757个。七是进一步谋划构建教育强市，继续推动了义务教育均衡发展和普通高中优质特色发展。本溪县创建基础教育强县（区）工作通过省政府验收。辽宁医药化工职业技术学院与辽宁医药职业学院一体化办学合作共建取得成果。八是县级公立医院改革完成阶段性任务。公共卫生均等化工作取得新成效。社区卫生服务中心建设纳入城市总体规划。农村妇女“两癌”免费检查1.3万人。九是帮助全市有需求的贫困肢体残疾人全部得到辅助器具救助服务。十是充分发挥市文化宫的演艺演出、会议会展、文化惠民等功能，举办大型演出125场，搭建“百姓联动大舞台”组织演出131场。市文联“文艺家之家”项目主体封顶。市文化艺术中心工程开工建设。特别是借助成功承办辽宁男篮CBA联赛，打出了“药都本溪”的品牌，叫响了“本是万物之根、溪乃四海之源，本本分分做人、点点滴滴做事”的本溪精神，集聚了“冬季到本溪来看球”的人气。火爆的赛事带动了火热的城市，小小的篮球场，正在成为展现本溪人民精气神、提升本溪产业和城市影响力的“大窗口”。

同时，本溪全域正式共享024区号，实现沈本通信一体化。积极开展安全生产大检查和隐患排查治理等专项整治行动，全市各类事故控制在省政府下达指标之内，未发生较大以上事故。深入推进领导干部接访下访等工作，有效化解了一批突出信访问题和矛盾隐患。全力推进平安本溪建设，继续积极探索“打防管控”一体化的管理模式，构建专业化、立体化的社会治安巡逻防控体系，较好地维护了社会和谐稳定。外事、侨务、对台、民族、宗教、人防、档案、史志、气象等工作也取得新的进展。

（十）扎实开展教育实践活动，政府自身建设得到加强

结合开展党的群众路线教育实践活动，政府系统作风建设立行立改，以市政府名义召开的会议和印发的文件、简报同比分别减少15%和8%，政府系统办公用房全部按规定标准整改完毕，制定并落实了市政府党组班子的“两方案一计划”。加强法治政府建设，制定了地方性法规草案和政府规章7件，办理行政复议案件226件。自觉接受市人大的依法监督和市政协的民主监督，高度重视并认真执行市人大及其常委会的决议决定，落实市政协提出的意见建议，全年办理人大代表建议479件、政协提案269件，问题解决率71.3%。公共资源交易平台平稳运行，全年成交金额71.4亿元，节约资金4.2亿元。深入落实党风廉政建设责任制，强化了行政监察和审计监督。

各位代表，过去的一年，我们遇到的困难比想象的要大，取得的成绩比预料的要好。这是全市上下在省委、省政府和市委的坚强领导下，在市人大、市政协的监督支持下，团结拼搏、攻坚克难、开拓创新、真抓实干的结果。在此，我代表市政府，向全市人民，各级人大代表、政协委员，各民主党派、工商联、无党派人士和人民团体，驻溪部队、武警官兵，中省直驻溪单位，向关心、支持和参与本溪发展建设的朋友们，表示衷心的感谢和崇高的敬意！

各位代表，当前，我市同全省、全国一样，经济发展进入增长速度的换档期、结构调整的阵痛期和前期刺激政策的消化期“三期叠加”的新常态，发展速度由过去两位数的高速增长转为个位数的中高速增长，经济结构处在深度调整、转型升级的关键时期，发展动力从要素驱动、投资驱动转向与创新驱动并行。在这种大环境、大背景下，我市经济发展受到的影响较大，2014年的生产总值、公共财政预算收入、固定资产投资等主要指标均有下降。分析原因：一是我市作为钢铁等传统产业比重较高的城市，增长速度下滑是结构调整的阶段性反映，体现出我市产业结构调整和转型升级任务仍然艰巨；二是受宏观环境影响，企业投资欲望不强，重大项目数量下降，新开工项目减少，传统产业的停产、半停产、亏损企业增加，经济增长的动力不足；三是面对经济发展新常态，政府服务效能需要进一步提升，机关作风建设有待于进一步加强。同时，经济社会发展中还存在财政收支矛盾进一步加剧、社会保障和融资偿债压力加大等问题。

二、2015年主要任务

2015年是全面深化改革的关键之年，是全面完成“十二五”规划的收官之年，也是为“十三五”起步打基础的重要一年。面对新常态，我们既清醒认识到当前面临的发展压力，更有决心和信心积极应对发展过程中遇到的困难和挑战。这种决心和信心来自于国家和省推出的一系列重大发展战略，来自于已经初见成效的“三都五城”新本溪这张好的蓝图，来自于在市委坚强领导下全市上下政通人和、齐心协力的良好氛围，来自于全市人民盼发展、谋发展的奋斗激情。只要我们集聚发展正能量，保持发展精气神，脚踏实地的坚持一张好的蓝图绘到底，就一定能够克服各种困难和挑战，在新一轮东北振兴中干在实处、走在前列！

今年市政府工作的总体要求是：把握新常态，抓住新机遇，坚持稳中求进工作总基调，坚持以提高经济发展质量和效益为核心，全力做好稳增长、促改革、调结构、惠民生、防风险各项工作，努力保持全市经济社会持续健康发展。

今年经济社会发展的主要预期目标是：全市生产总值增长6%左右；公共财政预算收入增长2%左右；固定资产投资增长6%左右；社会消费品零售总额增长11%左右；努力实现城乡居民收入与经济发展同步。

实现上述总体要求和目标任务，要重点做好以下工作：

第一，推动转型升级，坚定不移地抓好“三都”建设

进一步实施“163”发展战略，加快建设绿色钢都。围绕打造“中国本溪·绿色钢都”发展目标，全力建设6个涉钢产业基地，培育物流配送、科技创新、金融服务3个平台。一是继续深化服务本钢工作。落实市政府与本钢战略合作框架协议，发挥政府引导作用，创新服务方式，全力支持本钢“1+5”板块发展，继续推进钢地深度融合。二是加快建设6个产业基地。高端绿色钢材生产基地重点支持本钢冷轧高强钢项目建设。装备制造精品铸件基地重点推进烁丰铸管、金鸿机械制造、参铁集团等企业平稳健康运行。钢材精深加工基地重点扶持恒通轧辊、银龙预应力和金桥焊材项目。冶金资源综合利用基地重点发展利用矿渣微粉生产水泥、利用氧化铁红生产磁性材料等项目。绿色矿山资源开发基地重点推进思山岭铁矿和大台沟铁矿项目建设。新材料产业基地重点推进生产母合金、非晶带材产品项目。三是强力推进3个平台建设。重点服务本钢钢铁新材料研究院技术创新平台、钢都金融服务平台和彩北钢贸物流平台搭建工作。我们要通过大力建设绿色钢都，引领我市传统优势产业尽快实现转型升级，让绿色钢都展示本溪发展新形象！

推进规模化、国际化、城市化进程，加快建设中国药都。继续坚持产业为重、科技支撑、大学为要、产城融合的发展原则，进一步实施“173”发展战略。围绕实现“规模化”，全力服务存量企业投产达产、释放产能，鼓励企业引进三类以上新药、仿制药及独家品种，培育具有拉动作用的大企业、大项目。同时，以修正等龙头企业为支撑，打造健康食品产业园。丰富产业内涵，实施“北药”产业发展规划，打造“北药”品牌。推动产业升级，实施健康产业发展规划，构筑以高新区为核心，以本溪县、桓仁县为两翼，打造本溪全域药都的发展格局。围绕实现“国际化”，促进欧美、日本等知名医药企业与区内企业合资合作和兼并重组，服务开普医疗、大石药业等企业拓展国内外市场。继续加强“一心两园八平台”建设。发挥科技资金的杠杆作用，引进高水平的海外学子研发项目、团队以及品种。促进科技、金融与产业相融合，推进医疗健康产业基金的运营和投入。发挥好中国医科大学国际教育学院作用，推进药都优质教育链的国际化。围绕实现“城市化”，支持沈阳药科大学加快建设主校区和省级大学科技园；支持中国医科大学本溪校区全部投入使用、辽宁中医药大学本溪校区建设完工、辽宁医药职业学院一期工程主体封顶。积极开发温泉、教育、健康养生等特色地产。推进辽宁中医药大学附属沈本医院、盛京医院产后康复中心、台商总部及温泉谷等健康服务业项目运营发展和加快建设，加大基础设施、城市功能项目建设和公共服务投入力度，完善沈本新城的要素配置，不断增强药都集聚辐射力，让综合实力在全国生物医药产业集群排名前5位的中国药都位次前移！

规划“十百千万”旅游总体布局，加快建设中国枫叶之都。突出“春看百花夏吸氧，秋赏枫叶冬沐泉”的旅游特色，努力建设10个旅游业集聚区，发展100条沟域旅游，开发和标识1000座适合旅游的山峰，发展1万家农家乐，打造全时、全域、全产业链、全媒介的旅游度假目的地。继续实施“512”温泉旅游战略，重点推动温泉寺、汤沟等项目加快建设，打造省内领先、全国一流的温泉旅游小镇。在本溪水洞、东风湖和五女山等地大力开发温泉资源，建设“中国温泉之城”。加大特色旅游山庄、葡萄酒酒庄建设力度，积极开发老年旅游、医疗旅游等新业态旅游产品。丰富完善“枫红指数”，将本溪打造成“中国赏枫度假第一品牌”。整合旅游宣传资源，丰富宣传方式，推进“智慧旅游”工程，实现年有品牌节庆、季有特色产品、月有主题活动目标。积极引进主题公园、旅游购物、大型演艺项目，完成20个沟域旅游招商任务。加强游客综合服务中心等旅游基础设施建设，构建两县区域旅游环线，全力打造2个国家4A级景区。坚持市场导向抓好改革与规划引领，促进旅游业与生态农业、观光工业和医疗健康产业的跨界融合，努力完善产业链条，提升服务水平，使本溪成为全国知名的旅游度假目的地，让“中国枫叶之都”在海内外叫得更加响亮！

第二，突出稳定增长，千方百计抓好经济运行

（一）抓好财税和项目“两个重点”，打牢稳增长基础

以确保财税收入平稳增长为目标，继续组织好财政收支。加强收入分析和调度，抓好税源建设，完善税收征管，挖掘非税收入潜力，清理规范税收等优惠政策，确保应收尽收。努力做大市本级财政规模。结合贯彻新《预算法》，深入推进财政改革。完善全口径政府预算管理制度，进一步推进预决算公开。按照“借、用、还”一体化原则，加强政府债务管理；在城市基础设施建设、环境治理等领域，探索政府与社会资本合作机制（PPP）等模式。强化财政监督检查，严控一般性支出特别是“三公”经费支出，集中有限财力保经济增长、保结构优化、保民生和谐。

以承接落实新一轮振兴政策措施为统领，深入推进“项目年”活动。继续全力向政策、向市场、向环境要机遇，在做实溪湖和南芬搬迁改造项目基础上，再争取1-2个老工业区或独立工矿区搬迁改造项目纳入国家政策支持范围。继续大力开展“项目年”活动。坚持市政府领导分工负责制和“点对点、县区服务日”等机制，实现服务项目制度化。重点推进辽宁中部环线高速本溪段等项目开工建设，田桓铁路、观音阁输水工程等项目加快建设，沈丹客专等项目竣工通车；桓仁抽水蓄能电站等项目前期工作取得突破。同时，结合抢抓新一轮东北振兴机遇，编制完成“十三五”规划。

（二）调优“三次产业”，提供稳增长支撑

着眼“五个一批”，抓好工业经济。加强工业经济的调控调度和预警预测，全力做好服务企业工作，支持一批企业正常生产平稳运行、启动一批停产半停产企业、再投产达产一批工业技改项目、新开工和储备一批项目、支持一批小微企业提升为规模以上企业。深入实施工业“五项工程”。力争新增销售收入超5亿元企业10户、超10亿元企业3户。重点推进150个投资亿元以上工业项目。9个产业集群销售收入实现1200亿元。完成省下达的企业并购和淘汰落后产能目标任务。

突出重点产业，抓好服务业发展。按照“三高两点”目标，继续落实好服务业发展《四年行动计划》。房地产业重点开发需求导向型的养生、养老、旅游等特色地产和高品质、低密度、高端地产等复合型地产项目，促进房地产企业销售，推动房地产业健康发展。积极解决空间要素问题，严格实行净地出让，扩大土地储备规模，提高货币化安置比例。大力发展建筑业，采取有效措施，推动建筑业企业由生产商向服务提供商转型升级。商贸物流业以“两近”城市为依托，以“三都”建设为载体，加快重点物流园区建设，推动正远物流一期全面投入使用，本溪商贸冷链物流一期和华联物流配送中心等项目建成运营。推进建设一个现代化的大型农产品交易市场。加快电子商务与物流业的融合发展，引进阿里巴巴、京东等知名电商企业入驻本溪。金融业重点是继续引进品牌金融机构，大力发展和规范小额贷款公司、融资担保公司。创新和拓展金融业为政府融资的渠道。继续支持城商行改革，推进农信社改制。努力实现“五个一”目标，即全市银行贷款余额突破1000亿元、金融业税收夯实10亿元、1家地方银行利税总额突破5亿元、金融业税收占服务业税收比重超过10%、1户企业在主板上市。同时，结合“三都”建设发展会展经济，举办好钢博会、药交会等会展活动。继续以辽砚、剪纸、版画等为载体，大力发展文化产业。继续抓好商贸流通业，开展丰富多彩的主题促销活动，推动“夜经济”发展，促进消费增长。支持华联、大商等大型商业企业发展社区连锁生鲜超市，构建15分钟便民生活圈。

立足生态资源优势，发展特色现代农业。稳定完善农村土地承包关系，推进土地承包经营权确权登记颁证工作。规范引导农村土地经营权有序流转，加快培育新型农业经营主体。积极发展乡村休闲旅游农业，本溪县争取创建国家休闲农业与乡村旅游示范县。以“北药”产业发展为主导，积极发展智慧农业、绿色农业，桓仁县加快建设“北药”产业园区。两县要重点培育销售收入10亿元以上的农产品加工龙头企业。继续发展中药材、山野菜、食用菌等特色产业，抓好“米袋子”、“菜篮子”建设。加大农产品质量安全监管力度。推进建设“千万亩经济林”工程，发展各类产业基地10万亩。确保全市林权发证率达到97%以上。进一步完善小型农田水利灌溉工程。加大农田基本建设和耕地保护力度，努力提高耕地质量。

第三，实施创新驱动，积极稳妥地深化改革扩大开放

以创新驱动战略为引领，增强转型升级的内生动力。以高新区申报纳入国家自主创新示范区范围为契机，以树立区域化、全国化、全球化发展理念为引领，围绕生物医药、钢材深加工、优质铸造等优势和有潜力的产业链，谋划创建产业技术创新战略联盟。依托本钢和国家中成药两个国家级技术中心，发挥大型企业创新的骨干作用。依托入驻高校资源，建设沈阳药科大学上海绿谷学院、辽宁科技学院移动互联学院、“三都”研究所等载体，开展多种形式的产学研用合作，进一步激发中小企业的创新活力。加快发展科技服务业，全面加强与中科院沈阳分院合作，推动科技创新和科技成果在本溪转移转化，促进科技经济深度融合。切实发挥人才作为创新驱动战略关键因素的作用，今年要出台相关政策，针对急需的专业人才招录公职人员1000人，吸引高校毕业生2000人在本溪就业创业，鼓励农民市民化3000人进城就业创业，进一步优化城市人口结构，实现劳动力有序转移，努力形成创业创新的新局面。

依托智慧城市建设，发展新一代信息技术产业。大力推进信息惠民、宽带中国、信息消费国家示范（试点）城市建设，进一步提升城市宽带接入和城域网传输交换能力，推动全地域普及3G/4G和WLAN（WIFI）网络覆盖。推进信息化和工业化深度融合，促进云计算、大数据等广泛应用，为产业和企业转型升级提供支撑。以智慧城市建设为平台，运用跨界、整合、创新理念，在智慧交通、智慧医疗、智慧政务、智慧社区等领域实现突破，培育发展电子商务等新一代信息技术产业。

继续深入推进各项改革。着眼于转变政府职能，进一步简政放权。继续取消、下放与经济增长和就业创业密切相关的行政审批事项。全面清理政府部门行政职权，组织编制部门行政审批事项清单、权力清单和行政收费事项清单。以项目为牵引，再造审批流程，提高审批效率。通过落实政策，优化服务，充分发挥市场配置资源的决定性作用，为小微企业发展壮大降低门槛、扫除障碍，为非公有制经济快速发展营造良好环境。同时，深入实施政府机构改革，稳步推进事业单位改革。稳妥推进公务用车改革。改革完善公共资源交易市场管理体制、监督和运行机制。推进网上审批和便民服务平台建设。进一步深化国有企业和国有资产管理体制改革。推进“先照后证”和工商、税务、质监“三证合一”登记制度改革。实施城区居民用水阶梯水价制度改革。协调推进沈本一体化11个方面、26项具体工作落到实处。

继续扩大对外开放。进一步完善扁平化、矩阵式的投资促进推进体制，围绕“三都”建设，采取区域招商和产业招商相结合方式，继续开展“请进来、走出去”活动。拓展日韩、西欧和北美市场，深耕港台地区，盯住长三角、珠三角和京津冀，并针对县区产业集群和服务业集聚区发展重点，集中力量赴江苏、浙江、广东、福建等地进行定点招商、专题招商，搞好会展招商。巩固与央企、知名民企及沪企辽宁行对接成果，力争再引进一批重大项目。推动外经贸服务、海关和出入境检验检疫本溪办事处“三位一体”，提升对外开放服务水平，加强进出口基地建设，为外贸进出口提供持续支撑。以开放为导向，提高城市竞争力。

第四，发挥主体作用，大力发展县区经济

县区是工作主体，市政府是服务主体，要支持各县区在推进项目建设、组织财税收入、优化服务环境、打好“空间百日会战”等方面发挥工作主体作用。继续支持本溪县抓好人参铁和玻璃制品产业集群；重点推进温泉寺天著温泉小镇和汤沟国际温泉旅游度假区建设，确保温泉、滑雪、酒店项目早日运营。继续支持桓仁县提高健康食品及葡萄酒产业和包装印刷产业集群化水平；促进旅游业提档升级，推进桓龙湖景区申报国家生态旅游示范区，全方位塑造“中国北方国际养生城”的内涵和形象。继续支持平山区依托桥北钢材深加工产业园，全力做好本钢冷轧高强钢、金桥焊材、大台沟铁矿等重大项目的配套和服务工作，积极推进福耀集团硅石深加工、晋亿集团紧固件产业基地建设。继续支持明山区结合太子河风光带建设，完善两岸道路、管网等基础设施，加快牛心台地区整体改造。推进新材料产业园和明山现代服务业集聚区加快建设。继续支持溪湖区依托本溪湖资源综合利用产业园，壮大发展资源综合利用、钢材深加工产业；以“新溪湖·印象”等项目为载体，整体开发太子河北岸；推动火连寨地区融入沈本产业经济带。继续支持南芬区围绕做好“人参铁”文章，加快推进聚鑫达短流程等重点项目建设，提升铸件产业集群发展水平。服务思山岭铁矿项目建设，规划绿色矿业小镇。

第五，抓好城市建设管理和环境保护，着力打造生态宜居城市

推进李家山特色地产、站前城市综合体开发、胜利路三角地开发、太子河沿岸慢行系统等建设项目。开展城市精细化管理年活动。实施园林绿化提升工程，新建10个微广场；打造3条景观示范路；进一步提升望溪公园、儿童乐园、平顶山公园等公园管理水平。实施市容环境净化工程，开展拆除违建乱建、对主干路牌匾广告进行综合整治等工作，进一步改善市容市貌。

推进“创卫”、“创模”和创建生态市工作。做好省级卫生城市复核工作，对照新标准夯实“创卫”工作基础。继续实施“青山、碧水、蓝天”和“节能减排”工程，启动“净土工程”,编制《本溪市城市环境总体规划》，划定全市生态保护“红线”。深入开展污染物总量控制工作，整治城区低空污染，取缔禁燃区内10吨以下燃煤锅炉，完成国家、省下达的空气质量指标任务。落实饮用水源环境安全保护措施，保障全市污水处理厂稳定达标运行。全面启动废弃矿山治理。推进桓仁县申报国家生态环境保护与建设示范区、本溪县申报国家生态文明先行示范区。

坚持以人为本，突出规划引领，重点抓好两县县城、沈本新城、南芬新城以及草河口、华来镇等小城镇建设，加快推进全市新型城镇化进程。抓好宜居乡村建设，完成6个省级“宜居示范乡镇”、16个“宜居示范村”建设任务，推动57个村达到省级“宜居达标村”建设标准。

第六，保障改善民生，全力促进社会和谐稳定

实事务求实效，民生连着民心，今年市政府要一以贯之地办好十大类民生工程。一是实施积极的就业创业政策，重点促进高校毕业生、就业困难人员就业，全年实现城镇实名制就业4.7万人，城镇登记失业率控制在4.3%以内。二是继续扩大社会保险覆盖面，统筹提高各项社会保险待遇水平，确保企业离退休人员养老金按时足额发放。全面落实以城乡低保、精准扶贫为核心的各项社会救助政策，建立临时救助制度。三是确保完成省政府下达的保障性安居工程任务指标。努力完成平山甲楼项目征迁工作。四是改造街巷路100条；更新改造人行道20万平方米。有序开通主城区至桥北工业园区等多条公交线路。五是推动物业管理服务提档升级，业主大会筹备、组建率达90%以上。实施“暖房子”工程300万平方米，配套推进旧住宅区环境综合整治工程。六是依法解决2.2万户居民房屋未确权问题。继续加大居住区违法建筑管理整治力度。编制城市停车系统规划。七是谋划构建教育强市，推动义务教育均衡发展县（区）建设进程和普通高中的优质特色发展，积极构建现代职业教育体系。八是加快公共文化服务体系建设。完成市文化艺术中心主体工程。九是不断提升基层医疗机构服务能力和水平，稳步推进县级公立医院改革。继续推进食品药品监管体制改革，加大食品药品监管力度。十是继续承办好CBA联赛，借力CBA平台，促进CBA赛事与体育事业、体育产业、全民健身和旅游业、服务业等相关产业的融合发展。

同时，进一步抓好安全生产、信访稳定工作。守住“五个严防发生”的工作底线，全力推进平安本溪建设。加强信用体系建设。实施“六五”依法治市规划。支持军队和国防后备力量建设，努力实现全国双拥模范城“八连冠”。继续做好人防、外事、侨务、对台、民族、宗教、档案、史志、残联、气象等工作。

各位代表，面对2015年的新任务、新挑战，市政府将继续坚持一个“法”字，建设依法行政的人民政府。各级政府及工作人员要带头遵守宪法及法律法规，严格按照法定权限和程序行使权力、履行职责，不断提高法治意识和依法行政能力。要主动接受市人大法律监督、市政协民主监督、社会各界和人民群众监督。以全面启动网上办理为契机，进一步提高人大代表建议和政协提案办理的效率和质量。继续坚持一个“实”字，建设真抓实干的人民政府。执政为民，重在落实。要坚持心无旁骛、心底无私、心心相映、心甘情愿的工作境界，创业、创新、创意、创优地推动工作落实。要强化问题导向，从制约发展的重大问题、群众反映的难点热点问题入手，以目标倒逼进度、以督查倒逼落实，确保干一件事成一件事。继续坚持一个“廉”字，建设清正廉洁的人民政府。坚决反对“四风”，坚持勤俭办一切事业。进一步深化政务公开，让权力在阳光下运行。严格执行党风廉政建设责任制，落实“一岗双责”职责，努力营造风清气正、廉洁高效的政务环境。

各位代表，让我们在省委、省政府和市委的坚强领导下，把握新常态，抓住新机遇，为建设“三都五城”新本溪，为全市经济社会实现科学发展、可持续发展、包容性发展而努力奋斗！
